# Minimally invasive, endoscopic Achilles tendon reconstruction using semitendinosus and gracilis tendons with Endobutton stabilization

**DOI:** 10.1186/s12891-016-1099-3

**Published:** 2016-06-03

**Authors:** Tomasz Piontek, Paweł Bąkowski, Kinga Ciemniewska-Gorzela, Monika Grygorowicz

**Affiliations:** Orthopedic Department, Rehasport Clinic, Górecka 30, Poznan, 60-201 Poland; Research and Development Department, Rehasport Clinic, Górecka 30, Poznan, 60-201 Poland

**Keywords:** Achilles tendon, Neglected rupture, Achilles endoscopy, Achilles reconstruction

## Abstract

**Background:**

Plantaris tendon, peronus brevis tendon and flexor hallucis longus tendon augmentation, commonly used in Achilles tendon rupture, often lead to weakening of injured foot and they require the immobilization after the surgery. It is essential to develop the technique, which gives no such limitation and allows for immediate functional improvement.

**Methods:**

We present our method of minimally invasive, endoscopic Achilles tendon reconstruction using semitendinosus and gracilis tendons with Endobutton stabilization.

**Results:**

Posterolateral and posteromedial portals were made approximately 3 cm above the posterosuperior part of the calcaneus to clean the area of the Achilles tendon endoscopically. Then the hamstrings are harvested and prepared for the “Endobutton” system. A midline incision of the skin is performed approximately 1 cm above the posterosuperior part of the calcaneus to approach to the posterosuperior part of the calcaneus. Then under fluoroscopy the calcaneus was drilled through using K-wire. The distal end of the graft equipped with an Endobutton loop was entered into the drilled tunnel in the calcaneus. Later, 8 consecutive skin incisions are performed. Proximal ends of the graft were brought out through the native Achilles tendon reaching medial and lateral skin incisions. The final step was to transfer and tie the graft ends through the most proximal skin incision.

**Conclusions:**

This minimally invasive, endoscopic technique allows reconstruction of the Achilles tendon using semitendinosus and gracilis tendons with Endobutton stabilization and can be used in so-called “difficult”, resistant cases as a “salvage procedure”.

## Background

The Achilles tendon ruptures constitute a common clinical problem [1]. Changes that occur due to the aging process also increase exposure to possible damage [2]. The Achilles tendon rupture causes sudden and severe pain in the acute phase, and if left untreated, can cause muscle weakness, resulting in worsened physical functionality of the patient [3].

Rupture is defined as chronic if it is present at least for 4–6 weeks [4, 5]. Cases of chronic rupture of the Achilles tendon do not respond effectively to conservative treatment and therefore they require repair utilising graft [6]. The indications for surgical management of neglected Achilles tendon ruptures include weakness of the triceps surae complex, functional lengthening of the gastrocnemius–soleus complex, and an apropulsive gait [7]. Treatment of neglected the Achilles tendon ruptures, or re-ruptures, often involves considerable technical problems. The most common are: an enlarged gap (>3 cm) between the tendon ends, preventing end to end stapling, scarring of the tendon stumps and adjacent parts, shortening of the rear calf muscle groups, loss of muscle contractility forming the Achilles tendon and problems with wound healing [8]. Due to these reasons, the surgical treatment of chronic damages differs from the treatment of acute Achilles tendon damage.

It has been confirmed that open technical procedures for the treatment of ruptures of the Achilles tendon lead to postoperative wound complications because of the fragility and limited vascularization of the skin [9], and also they can increase the risk of the infection and morbidity [10]. Thus, minimally invasive techniques have been developed [11–16], however they are technically demanding [17].

Typical methods of repairing neglected Achilles tendon damage are augmentation of the plantaris tendon, peroneus brevis tendon and flexor hallucis longus tendon. Tendon transfer techniques are being commonly used, yet often result in permanent functional complications. Flexor hallucis longus transfer involves a decrease of hallux flexion strength [14, 18]. The loss of foot eversion strength associated with the transfer of the peroneus brevis tendon is little, however, subjective reduction in foot strength may occur [15, 16]. Use of turn down flaps is also widely used in neglected Achilles tendon ruptures. In cases of large gap (more than 3 cm) it requires large interference in the proximal stump, which often lacks in quality and needs additional reinforcement [19]. Additionally patient is exposed to wound healing problems due to large skin incision and Achilles tendon exposure.

## Methods

We present our method of minimally invasive, endoscopic Achilles tendon reconstruction for patients with neglected tendon rupture, with end gap over 3 cm. In our technique we use semitendinosus and gracilis tendons transfer with Endobutton stabilization.

### Surgical technique

The surgery is routinely performed under spinal anesthesia. The patient lay in prone position with the pneumatic tourniquet applied at the midthigh at a fixed pressure of 250 mmHg, in order to obtain ischemia within the surgical field.

### Achilles endoscopy

**Fig. 1:**
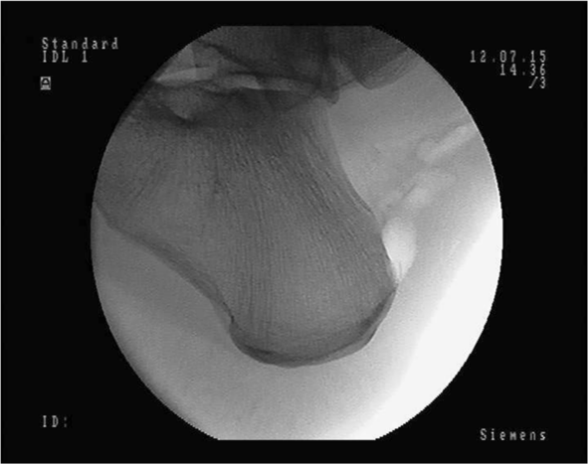
X-ray control of the shape of the calcaneus

The first stage of the operation is endoscopic cleaning of the Achilles tendon. For this purpose, posterolateral and posteromedial portals are performed approximately 3 cm above the posterosuperior part of the calcaneus. By use of video control, the Achilles tendon bursa is visualized and then it is removed. The area of the Achilles tendon is cleaned from adhesions and pathological tissues. Within such approach the attachment area of the Achilles tendon is cleaned. The next step is to remove excessive bone from the heel using a bone shaver and to control the shape of the calcaneus on the fluoroscopy screen (Fig. 1).

### Graft preparation

An approximately 3 cm incision is made just below the knee on the inside and top part of the tibia to make the semitendinosus and gracilis tendons visible. Harvested grafts are cleaned and hemmed with an Ethibond 2 thread (Ethicon, USA). Afterwards the hamstring graft is prepared for the Endobutton system (Smith & Nephew, USA). Both tendons are grouped together and folded in half creating a bundle of 4 (the length of about 10 cm, from 7 mm to 9 mm thick, provided with an Endobutton loop system). The length of the loop is chosen according to the total length of the calcaneus bone tunnel to obtain an intracalcaneal graft length of a minimum 1.5 cm.

### Preparation of the tunnel in the calcaneus

**Fig. 2:**
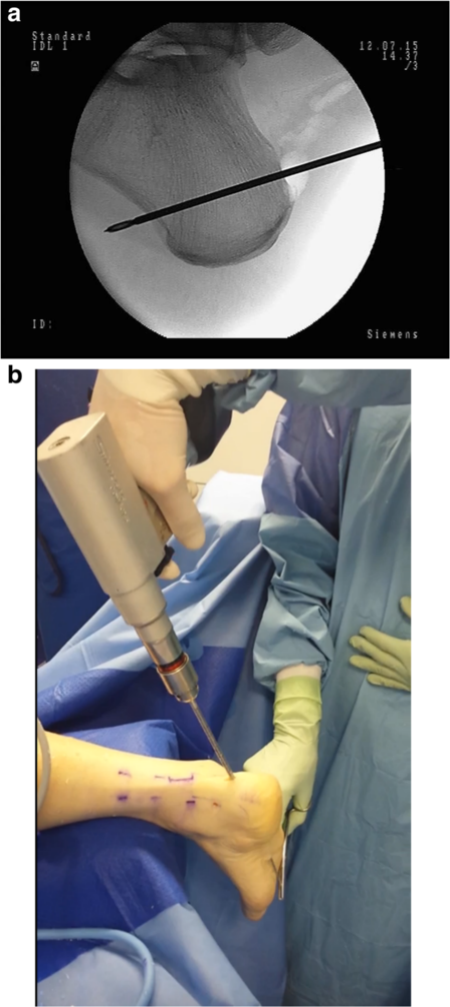
**a**. X-ray control of K-wire placement. **b**. Calcaneus drilling using skin incision No 1

**Fig. 3:**
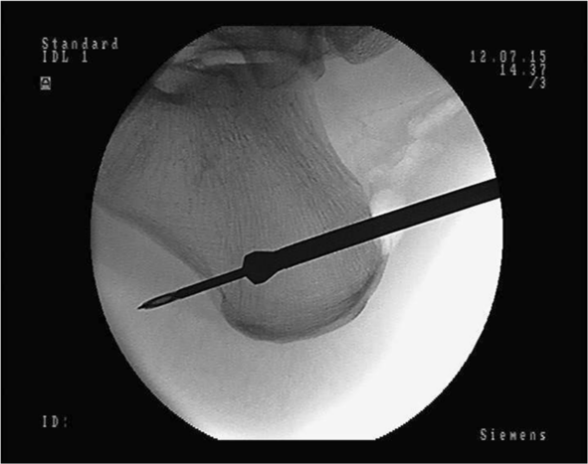
X-ray control of calcaneus drilling

While the assistant is preparing the graft, the surgeon is performing a tunnel in the calcaneus. A midline incision of the skin is performed approximately 1 cm above the posterosuperior part of the calcaneus, and then expanded by use of a Pean clamp. This portal (skin incision number 1) is used as a surgical approach to the posterosuperior part of the calcaneus. Then, under fluoroscopy, the calcaneus is drilled through using K-wire so that the distal end of the tunnel is located anterior to the foot plantar fascia attachment. (Fig. 2a, b). Such a location of the tunnel enables proper biomechanical functioning of the reconstructed tendon (the place of Endobutton fixation coincides with the final attachment of the Achilles tendon), and prevents irritation of the plantar fascia. A thick layer of the posterior-bottom calcaneus cortex provides sufficient durability for the Endobutton. The next step of the procedure is drilling by use of a 4.5 mm drill, the measurement of the tunnel length, and its extension to adequate size (depending on graft size), maintaining the distal cortex of the heel in reliance to the corresponding Endobutton fixation (Fig. 3).

### Skin incisions

**Fig. 4:**
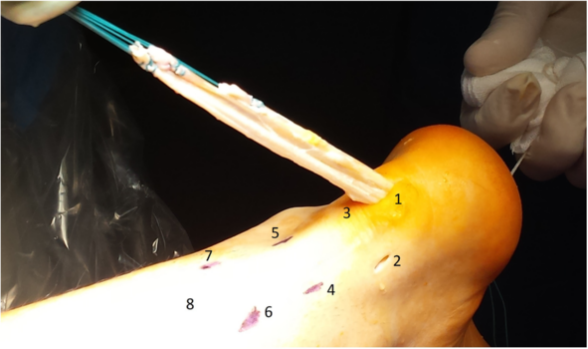
Skin incisions and first step of the Achilles tendon reconstruction

After appropriate preparation of the bone tunnel in the calcaneus, 7 consecutive skin incisions are made as shown in the scheme (Fig. 4). The most proximal skin incision (number 8) serves as the final portal, through which the threads are tied. Skin incision number 1 is used as a surgical approach to the posterosuperior part of the calcaneus. Lateral skin incisions number 2, 4, 7 and the medial incisions number 3, 5, 7 are used to interleave the graft, analogous to the method of percutaneous the Achilles tendon suturing [8, 18] (Fig. 4).

### Restoring the Achilles tendon continuity

**Fig. 5:**
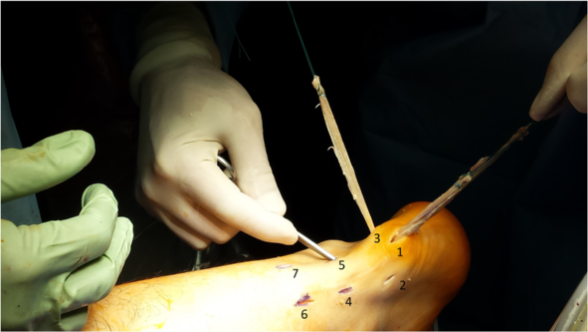
Scheme of the Achilles tendon reconstruction

**Fig. 6:**
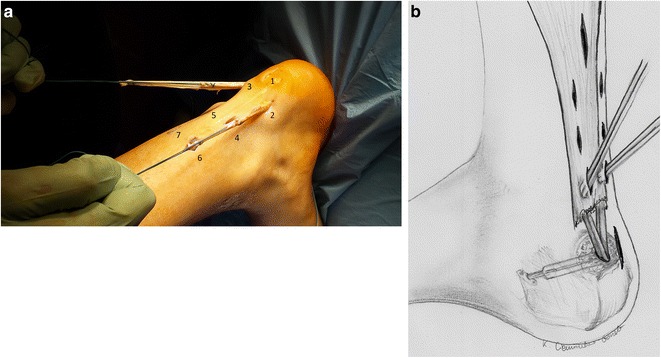
**a**. Scheme of the Achilles tendon reconstruction. **b**. A schematic drawing showing the procedure

**Fig. 7:**
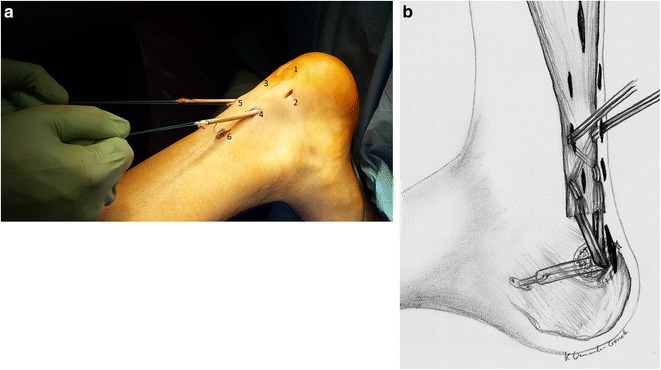
**a**. Scheme of the Achilles tendon reconstruction. **b**. A schematic drawing showing the procedure

**Fig. 8:**
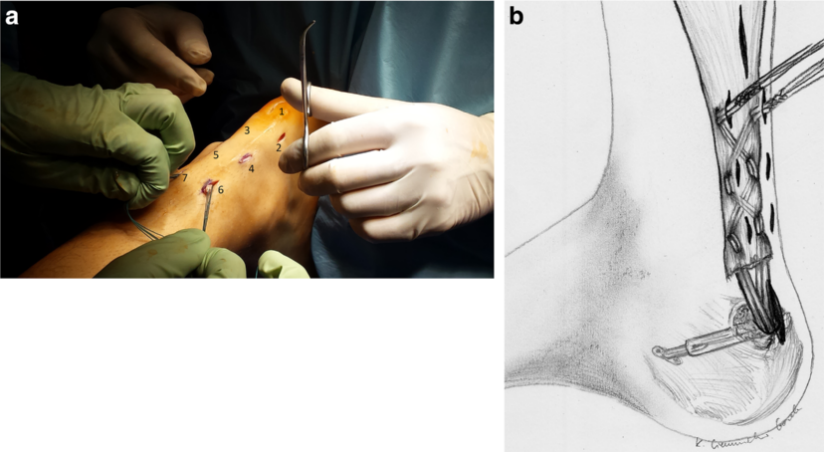
**a**. Scheme of the Achilles tendon reconstruction. **b**. A schematic drawing showing the procedure

**Fig. 9:**
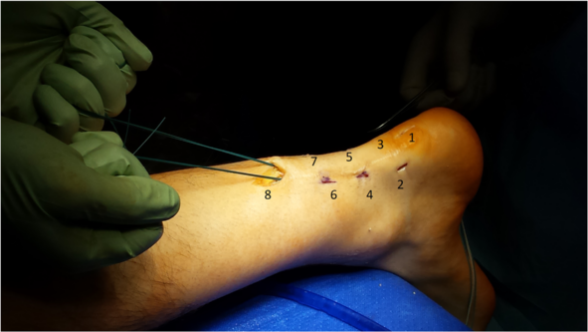
Scheme of the Achilles tendon reconstruction

**Fig. 10:**
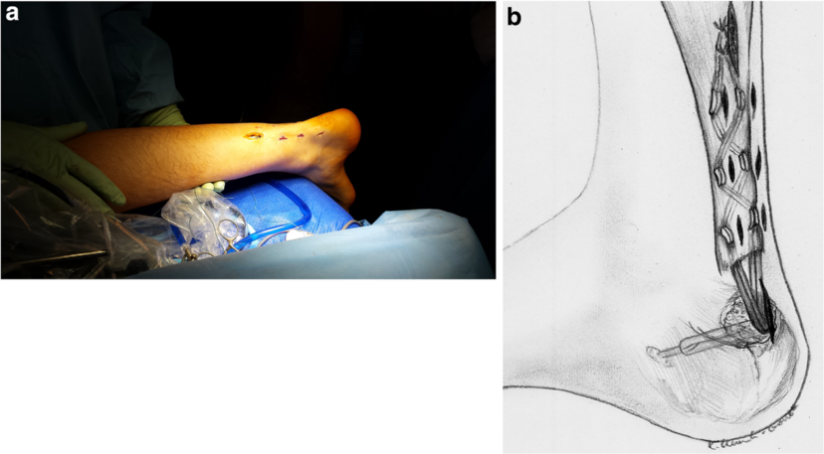
**a**. Achilles tendon after minimally invasive, endoscopic Achilles tendon reconstruction using semitendinosus and gracilis tendons with Endobutton stabilization. **b**. A schematic drawing showing the procedure

Using the number 1 skin incision, the distal end of the graft equipped with an Endobutton loop is entered into the drilled tunnel in the calcaneus (Fig. 4). The correct position of the Endobutton loop is controlled using flouroscopy. According to the percutaneous Achilles tendon suturing technique [13], proximal ends of the graft are brought out through the native Achilles tendon reaching medial and lateral skin incisions. This way, proximal and distal ends of the damaged Achilles tendon are brought closer and both, proximal and distal, the Achilles stumps are connected with the graft. The diagram presents the graft passage order (Figs. 5, 6a, b, 7a, b, 8a, b and 9). The final step is to transfer and tie the graft ends through the number 8 skin incision (Fig. 10a, b). Threads are tied together after prior tension in plantar flexion of about 20°. Correctness of the Achilles tendon tension is controlled by palpation and the Thompson test, which has been confirmed as the strongest diagnostic test of all measures for assessment the Achilles Tendon injuries [20].

### Suturing wounds

After releasing the tourniquet and hemostatic control, wounds are sutured. Afterwards, a Jones dressing is performed with 10° of plantar flexion.

### Postoperative care

Patients are discharged on the day after surgery after being instructed by a physiotherapist about using crutches and rehabilitation for the first 2 weeks. We do not use any immobilisation neither orthosis. Thromboprophylaxis is provided with Fraxiparine (nadroparin calcium, GlaxoSmithKline) 0.6 ml administered subcutaneously once a day. Partial weight bearing is allowed immediately after the surgery and full weight bearing after 2 weeks if tolerated. There are no limitations in the active range of motion of the operated foot. A follow-up postoperative x-ray is obtained at 2 weeks post-surgery.

## Discussion

The main findings of the present study is that our new method of minimally invasive, endoscopic Achilles tendon reconstruction using semitendinosus and gracilis tendons with Endobutton stabilization can be a treatment option for patients with neglected Achilles rupture with the end gap over 3 cm.

Treating neglected Achilles tendon rupture is challenging, and there is still a scientific debate over the surgical approach (open or percutaneous), suture repair method and suture type [21]. Conventional operative treatments for chronic damages of the Achilles tendon are plantaris tendon, peroneus brevis tendon and flexor hallucis longus tendon augmentation [13–16]. Turn down flaps is also widely used in neglected Achilles tendon [8]. All of these techniques use a single longitudinal incision for exposure. Following these procedures, complications, especially wound breakdown and infection, are not infrequent. They are probably related to the paucity of the soft tissue vascularity, and they may require plastic surgical procedures to cover significant soft tissue defects [8]. Moreover, following these procedures, complications, especially foot strength weakening is observed [13–16]. In our opinion we should avoid to weaken, already injured and weakened foot, and surgeons should try to use grafts harvested anatomically placed away from the foot. That is why, following this concept of using hamstring graft for chronic Achilles reconstructions seems to be the ideal resolution.

We perform this type of minimally invasive reconstruction in patients with neglected Achilles tendon rupture with end gap over 3 cm, neglected partial damage resulting from Achilles tendon dysfunction and in cases who failed of previous conservative and surgical treatment (Table 1). This technique derived from the open technique and is used by the author in so-called “difficult cases” as a “salvage procedure”. We decided to upgrade our open technique into minimally invasive to minimalize the wound healing problems and to reduce the rate of infection, which we challenged in our clinical experience, and which were also observed by other authors [19].

**Table 1:** Indications and contraindications for minimally invasive, endoscopic Achilles tendon reconstruction using semitendinosus and gracilis tendons with Endobutton stabilization

Indications - neglected Achilles ruptures with end gap > 3 cm - neglected partial damage (>50 %) resulting from Achilles tendon dysfunction - failure of previous conservative and surgical treatment	
Contraindications - metabolic disorders - infections	

Minimally invasive technique of Achilles reconstruction limits the risk of damaging surrounding tissue when it is compared to open techniques [22]. Suturing the Achilles tendon with the Bunnel suture is widely used [13, 23]. Such approach provides adequate tensile strength and it can be implemented into percutaneous technique. The use of the hamstring autograft was proven to be safe and effective in reducing autoimmune reactions [23–26].

The advantage of this technique is that it allows performing a reconstruction using semitendinosus and gracilis tendons with Endobutton stabilization in a minimally invasive way (Table 2). Eight skin incisions, definitely smaller comparing to the size of incisions in technique with two skin cut [19], allow for more appropriate treatment and preserving skin integrity. Using semitendinosus and gracilis graft does not influence negatively on strength and power of the foot. Our technique provides sufficient biomechanical conditions to fast, post-operative foot functional improvement by harvesting the tendons that are placed away from the ankle. Post-operative treatment is conducted without the need for immobilization of the ankle or ankle orthosis. It allows to minimalize the gait pattern disturbance, and patients are able to walk normally very fast, which is great advantage. This technique is not very difficult to perform. Surgeon should be able to perform tendon harvesting, percutaneous suturing and heel endoscopy. More pitfalls of minimally invasive, endoscopic Achilles tendon reconstruction using semitendinosus and gracilis tendons with Endobutton stabilization are provided in Table 3.

**Table 2:** Advantages of minimally invasive, endoscopic Achilles tendon reconstruction using semitendinosus and gracilis tendons with Endobutton stabilization

Advantages - fast and simple procedure - minimal risk of wound healing complications - no weakening of injured foot - minimal risk of donor site morbidity - very fast functional improvement - no need of immobilization, no need of orthosis use

**Table 3:** Pitfalls in minimally invasive, endoscopic Achilles tendon reconstruction using semitendinosus and gracilis tendons with Endobutton stabilization

Pitfalls - entry point in calcaneal tunnel should be properly localized it minimize the risk of calcaneal fracture and clash of Achilles tendon with shoes - distal end of calcaneal tunnel should be located anterior to plantar fascia attachment, where the posterior-bottom calcaneus cortex is thick - be aware of sural nerve damage (we advise larger skin incision number 8 to visualize the nerve)	

We acknowledge that this paper is a technical note, and no data about clinical results of our patients are presented. However, we would like to inform that our preliminary results are very encouraging and we plan to analyze the long-term outcomes of this technique. This will be the subject of future research projects.

## Conclusion

This technique allows for minimally invasive, endoscopic reconstruction of Achilles tendon using semitendinosus and gracilis tendons with Endobutton stabilization, and it can be a treatment option for patients with neglected Achilles tendon rupture with end gap over 3 cm, and in cases who failed of previous conservative and surgical treatment.
